# Association of autosomal dominant polycystic kidney disease (ADPKD) with cardiovascular disease and mortality in patients with treated kidney failure

**DOI:** 10.1186/s12882-025-04185-1

**Published:** 2025-09-29

**Authors:** Julia Jefferis, Andrew J. Mallett, Gopi Rangan, Yeoungjee Cho, Andrea K. Viecelli, Venkat Vangaveti, David W. Johnson, Carmel M. Hawley

**Affiliations:** 1 Department of Nephrology, Mater Health Service, Brisbane, Australia; 2 Department of Renal Medicine, Townsville University Hospital, Douglas, QLD Australia; 3 Department of Renal Medicine, Westmead Hospital, Western Sydney Local Health District, Westmead, Australia; 4 Department of Kidney and Transplant Services, Princess Alexandra Hospital, Brisbane, Australia; 5 College of Medicine and Dentistry, James Cook University, Townsville, QLD Australia; 6 Townsville Institute of Health Research and Innovation, Townsville University Hospital, Douglas, QLD Australia; 7 Australasian Kidney Trials Network, University of Queensland, Brisbane, Australia; 8 Translational Research Institute, Brisbane, Australia

**Keywords:** ADPKD, Cardiovascular disease, Dialysis, Kidney failure, Transplant

## Abstract

**Introduction:**

Autosomal dominant polycystic kidney disease (ADPKD) is a multisystem syndrome associated with significant morbidity and mortality, particularly kidney failure. This study sought to evaluate mortality and transplant outcomes in Australian and New Zealand patients with ADPKD commencing kidney replacement therapy (KRT).

**Methods:**

A retrospective review of all patients with kidney failure over 18 years of age commencing KRT between 1963 and 2020, stratified across eras, using Australia and New Zealand Transplant (ANZDATA) Registry data. People with ADPKD were compared to those with other causes of kidney failure (non-ADPKD). The primary outcome was mortality on dialysis. Secondary outcomes included post-kidney transplant patient survival and graft survival (both death-censored and with death as a competing risk). Outcomes were compared using multivariable Cox proportional hazards models.

**Results:**

During the study period, 63,875 patients commenced KRT, including 2,466 (3.9%) with ADPKD. Compared to patients without ADPKD, those with ADPKD had generally fewer comorbidities at KRT initiation. ADPKD was independently associated with a lower risk of mortality on dialysis (adjusted hazard ratio [aHR], 0.71, 95% CI 0.67–0.75, *p* < 0.001). Causes of death were similar between the ADPKD and non-ADPKD cohorts including for cardiovascular disease (27.5% vs. 27.6%, respectively), infection (7.9% vs. 8.5%) and cancer (3.4% vs. 3.5%). Kidney transplant recipients had fewer comorbidities across both cohorts, with a similar incidence of coronary artery disease (ADPKD 7.2% vs. non-ADPKD 8.0%). Compared with non-ADPKD, ADPKD was associated with a similar mortality risk in kidney transplant recipients (aHR, 0.96, 95% CI 0.88–1.05) and slightly improved graft survival (aHR, 0.87; 95% CI 0.77–0.97, *p* < 0.05), although there was no difference in death-censored graft survival (aHR, 0.92, 95% CI 0.79–1.07).

**Conclusions:**

Compared to other causes of kidney failure, ADPKD is associated with better patient survival on dialysis, similar patient survival post-kidney transplantation and similar death-censored graft survival.

## Introduction

Autosomal Dominant Polycystic Kidney Disease (ADPKD) is a multisystem disease associated with significant cardiovascular and kidney morbidity and mortality, including a high prevalence of kidney failure in a young population [1]. Understanding disease trends and factors associated with adverse outcomes is fundamental, especially given the large burden of kidney failure and cardiovascular disease in Australia and New Zealand [2–4]. 

ADPKD is a heritable disorder which is one of the most common causes of kidney failure globally. ADPKD arises due to mutations in *PKD1* or *PKD2*, inherited in an autosomal dominant manner. ADPKD(*PKD1*) is associated with earlier age of onset and more severe forms of disease compared to ADPKD(*PKD2*) [5]. Population studies highlight the significant morbidity associated with ADPKD, as more than half of patients with ADPKD either die or require kidney replacement therapy (KRT) by 53 years of age [6]. Factors associated with reduced kidney function in ADPKD include ADPKD(*PKD1*), male gender, age of diagnosis, hypertension and gravidity ≥three, although these factors are not consistent in all studies [6–8]. In Australia and New Zealand, the incident rate of KRT for kidney failure due to ADPKD increased 3.2-fold between 1970 and 2010, which may have been due to improved KRT availability and accessibility. During the same time period, five year survival increased from 52 to 85%, although this improvement was less than that seen in the non-ADPKD population and may have reflected improvements in care including cardiovascular risk reduction [9]. Understanding morbidity and mortality trends in the ADPKD cohort is important to understand risk guide treatment strategies.

ADPKD impacts morbidity in a younger population including cardiovascular disease, hypertension, cyst complications, diverticulitis and kidney failure [10]. Cardiovascular disease is highly prevalent, with 21% of patients receiving dialysis across Australia and New Zealand dying from cardiovascular disease, including those with ADPKD [11, 12]. Cerebrovascular disease has increased in the ADPKD population, although associated deaths have reduced [9, 13, 14]. Morbidities in ADPKD cohorts may reflect disease burden associated with kidney failure, similar to others with kidney disease, but may also have disease specific morbidities.

This observational, retrospective, descriptive cohort study aimed to compare mortality and transplant outcomes between adult patients with and without ADPKD commencing KRT in Australia and New Zealand.

## Methods

### Study population

Data for adult patients (> 18 years) commencing KRT between 1963 and 2020 who were enrolled in the Australian and New Zealand Dialysis and Transplant (ANZDATA) Registry was obtained for this study [4]. Patients with primary kidney disease attributed to ADPKD were compared to the remainder of the cohort with an alternative primary diagnosis for kidney disease. Collection and analysis of ANZDATA Registry data were approved by the Metro South Ethics Human Research and Ethics Committee (HREC/2022/QMS/84918).

### Data collection

Baseline characteristics were evaluated at the initiation of dialysis or, if pre-emptively transplanted, at the time of transplant. Baseline demographics were collected including age, sex, racial origin, KRT era, body mass index, state, first KRT, follow-up years and, in a contemporary cohort (1996–2020), medical comorbidities including diabetes mellitus, hypertension, coronary artery disease, peripheral arterial disease, cerebrovascular disease, chronic lung disease and smoking status (current, former, never).

### Clinical outcomes

The primary outcome was patient survival on dialysis. Secondary outcomes were post-kidney transplant patient survival and graft survival (including death-censored and with death as a competing risk).

### Statistical analyses

Only patients who had dialysis as the first KRT were included in the patient survival on dialysis analysis. Kidney transplant survival was only considered for first kidney transplant recipients. Data were censored for death or end of study date (31/12/2022) or loss to follow up date. Results were expressed as frequencies and percentages for categorical variables, mean ± SD for continuous normally distributed variables with level of significance for comparisons between two groups reported based on Levene’s test for equality of variances. Baseline variables were summarised using counts and percentages. They were then assessed by χ^2^ tests of independence. Continuous variables were assessed with Students t test or one way Analysis of Variance (ANOVA). Univariable and multivariable Cox proportional hazards models were used to estimate the association between exposures and outcome variables (age, gender, smoking status, BMI, racial origin, medical comorbidities, dialysis modality, dialysis vintage, donor source, donor age and transplant vintage). Hazard ratios (HRs) and 95% confidence intervals (CIs) were calculated for each variable. Graft survival was examined using multiple approaches, including time to graft loss (including death), time to death-censored graft loss and in competing risk regression whereby death was a competing event. Partial (Schoenfeld) residual method to test for non-proportionality of the cox models. Time zero was taken at initiation of KRT, either transplant or dialysis. Results were considered statistically significant if *p* < 0.05. Only complete cases were included in the analyses. Missing data is reported in Tables 1 and 2. All analyses were conducted in SPSS software (IBM Corp. Released 2021. IBM SPSS Statistics for Windows, Version 28.0. Armonk, NY: IBM Corp).

**Table 1:** Baseline characteristics of the dialysis cohort

Characteristics	Non-ADPKD *N* = 61,409 (96.1%)	ADPKD *N* = 2466 (3.9%)	*P*-value
**Age (years)**	62.59 ± 13.8	61.66 ± 12.1	< 0.001
< 20	321 (0.5)	11 (0.4)	< 0.001
20–39	3775 (6.1)	89 (3.6)	
40–59	18,038 (29.4)	869 (35.2)	
60–79	34,380 (56.0)	1390 (56.4)	
80+	4895 (8.0)	107 (4.3)	
**Gender**			
Female	24,985 (40.7)	1124(45.6)	< 0.001
Male	36,424 (59.3)	1342 (54.4)	
**Smoking status**			
Never	24,547(40.0)	1051 (42.6)	< 0.001
Former	23,446 (38.2)	807 (32.7)	
Current	7706 (12.5)	256 (10.4)	
Missing	5710 (9.3)	352 (14.3)	
**BMI** (kg/m^2^)	28.4 ± 7.4	26.90 ± 6.5	< 0.001
**Ethnicity**			
White	32,387 (52.7)	1645 (66.7)	< 0.001
First Nations Australian	5752 (9.4)	23 (0.9)	
Māori	4488 (7.3)	51 (2.1)	
Asian	4720 (7.7)	111 (4.5)	
Other	13,683 (22.3)	629 (25.5)	
Missing	379 (0.6)	7 (0.3)	
**eGFR**	7.2 ± 5.1	7.1 ± 6.8	0.39
**Comorbidities at KRT entry**			
Chronic lung disease	8612 (15.3)	211 (9.9)	< 0.001
Coronary artery disease	21,688 (38.3)	552 (25.8)	< 0.001
Peripheral vascular disease	12,421 (22.1)	173 (8.1)	< 0.001
Cerebrovascular disease	7351 (13.1)	243 (11.4)	0.027
Cancer	9865 (16.2)	327 (13.4)	< 0.001
Diabetes	31,039 (54.4)	248 (11.6)	< 0.001
**Dialysis modality**			
Haemodialysis	43,830 (71.4)	1632 (66.2)	< 0.001
Peritoneal dialysis	17,579 (28.6)	834 (33.8)	
**Dialysis vintage**			< 0.001
1989–1998	13,131 (21.4)	770 (31.2)	
1999–2008	17,845 (29.1)	596 (24.2)	
2009–2018	23,525 (38.3)	798 (32.4)	
2018–2021	6908 (11.2)	302 (12.2)	
**Mortality rate**			
1 year mortality	20,008 (32.6)	567 (23.0)	< 0.001
3 year mortality	37,137 (60.5)	1193 (48.4)	< 0.001
5 year mortality	47,573 (77.5)	1651 (67.0)	< 0.001
**Cause of mortality**			< 0.001
Cardiovascular	16,934 (27.6)	679 (27.5)	
Infection	5234 (8.5)	194 (7.9)	
Withdrawal	11,132 (18.1)	408 (16.5)	
Cancer	2174 (3.5)	83 (3.4)	
Other	9964 (16.2)	319 (12.9)	
Not applicable (did not die)	15,971 (26.0)	783 (31.8)	

Abbreviations: BMI = body mass index, Significance level: *<0.05, **<0.01, ***<0.001

**Table 2:** Baseline characteristics of the transplant cohort

Characteristics	Non- ADPKD *N* = 26,357 (87.9%)	ADPKD *N* = 3630 (12.1%)	*P*-value
**Recipient Age (years)**	40.97 ± 15.44	49.85 ± 10.3	< 0.001
< 20	2503 (9.5)	62 (1.7)	< 0.001
20–39	9116 (34.6)	369 (10.2)	
40–59	11,672 (44.3)	2630 (72.5)	
60–79	3065 (11.6)	569 (15.7)	
80+	1 (0.003)	0 (0)	
**Recipient Gender**			< 0.001
Female	10,091 (38.3)	1600 (44.1)	
Male	16,266 (61.7)	2030 (55.9)	
**Recipient Smoking status**			< 0.001
Never	12,117 (46.0)	1805 (49.7)	
Former	6105 (23.2)	975 (26.9)	
Current	2166 (8.2)	203 (5.6)	
Missing	5969 (22.6)	647 (17.8)	
**Recipient BMI (**kg/m^2^)	25.6 ± 6.2	26.1 ± 4.8	< 0.001
**Recipient Ethnicity**			< 0.001
White	16,575 (62.9)	2573 (70.9)	
First Nations Australian	843 (3.2)	13 (0.4)	
Maori	792 (3.0)	27 (0.7)	
Asian	2572 (9.8)	155 (4.3)	
Other	5351 (20.3)	853 (23.5)	
Missing	224 (0.8)	9 (0.2)	
**eGFR**	7.04 ± 6.63	7.1 ± 5.76	0.54
**Recipient comorbidities**			
Chronic lung disease	812 (3.7)	76 (2.4)	< 0.001
Coronary artery disease	1745 (8.0)	225 (7.2)	0.12
Peripheral vascular disease	925 (4.2)	47 (1.5)	< 0.001
Cerebrovascular disease	556 (2.5)	118 (3.7)	< 0.001
Cancer	1459 (5.6)	263 (7.3)	< 0.001
Diabetes	4592 (20.3)	107 (3.3)	< 0.001
**First KRT modality**			
Haemodialysis	15,576 (59.9)	2275 (62.7)	< 0.001
Peritoneal dialysis	8069 (30.6)	894 (24.6)	
Pre-emptive transplant	2512 (9.5)	461 (12.7)	
**Dialysis vintage (yr**,** mean**,** SD)**			< 0.001
1989–1998	11,909 (45.2)	1289 (35.5)	
1999–2008	6250 (23.7)	990 (27.3)	
2009–2018	7614 (28.9)	1240 (34.2)	
2018–2021	584 (2.2)	111 (3.1)	
**Cold ischemia time (hr**,** mean**,** SD)**	10.27 ± 7.2	10.28 ± 6.9	0.94
**Donor age**			< 0.001
< 20	3508 (13.8)	405 (11.3)	
20–39	8085 (31.8)	881 (24.7)	
40–59	10,232 (40.2)	1660 (46.5)	
60 & above	3624 (14.2)	625 (17.5)	
**Donor source**			
Deceased	19,238 (73.2)	2622 (72.3)	0.26
Live donor	7046 (26.8)	1004 (27.7)	
**Transplant era**			< 0.001
1963–1998	10,542 (40.0)	1118 (30.8)	
1999–2008	5614 (21.3)	839 (23.1)	
2009–2018	7255 (27.5)	1245 (34.3)	
2018–2021	2946 (11.2)	428 (11.8)	
**Mortality rate**			
1 year mortality	1198 (4.5)	148 (4.1)	0.21
3 year mortality	3153 (12.0)	439 (12.1)	0.83
5 year mortality	5659 (21.5)	776 (21.4)	0.91
**Cause of mortality**			< 0.001
Cardiovascular	4082 (15.5)	474 (13.1)	
Infection	1978 (7.5)	234 (6.4)	
Withdrawal	784 (3.0)	104 (2.9)	
Cancer	1818 (6.9)	318 (8.8)	
Other	2374 (9.0)	311 (8.6)	
Missing	15,321 (58.1)	2189 (60.3)	
**Graft failure rate**			
1 year graft failure	3144 (11.9)	295 (8.1)	< 0.001
3 year graft failure	4001 (15.2)	354 (9.8)	< 0.001
5 year graft failure	4747 (18.0)	413 (11.4)	< 0.001
**Cause of graft failure**			< 0.001
Rejection (acute + hyperacute)	35.151607 (19.6)	168 (24.6)	
Chronic allograft nephropathy	4244 (51.7)	335 (8.5)	
Vascular	576 (7.0)	58 (8.5)	
Technical	221 (2.7)	20 (2.9)	
Glomerulonephritis	473 (5.8)	7 (1.0)	
Non-compliance	267 (3.3)	7 (1.0)	
Other	825 (10.0)	89 (13.0)	
**Disease in graft kidney**			0.013
BK virus nephropathy	453 (59.0)	66 (68.8)	
De novo glomerulonephritis	199 (25.9)	26 (27.1)	
Glomerulonephritis in graft	116 (15.1)	4 (4.2)	

Abbreviations: BMI = body mass index, Significance level: *<0.05, **<0.01, ***<0.001

## Results

### Population characteristics

#### Dialysis cohort

Between 1963 and 2020, a total of 63,875 patients commenced KRT, of whom 2,466 (3.9%) had a primary diagnosis of ADPKD. Baseline characteristics of the KRT dialysis group are reported in Table 1. Compared to the non-ADPKD cohort, patients with ADPKD were more likely to commence dialysis between 40 and 59 years, with lower rates of dialysis initiation at the extremes of age (20–39 and over 80 years). Patients with ADPKD were also less likely to be current smokers, First Nations Australian, Māori, obese or medical comorbidities of diabetes, cardiovascular disease, peripheral vascular disease, cancer and diabetes at KRT initiation, although the proportion with cerebrovascular disease was similar (Table 1).

### Transplant cohort

A total of 29,987 patients underwent kidney transplantation, of whom 3,630 (12.1%) had a diagnosis of ADPKD. Their baseline characteristics are reported in Table 2. Comparative rates of deceased and live donation were similar. Compared to the non-ADPKD group, patients with ADPKD were more likely to be older and White, and less likely to be First Nations Australian, Māori, or have comorbidities, except for a similar proportion of coronary artery disease and a higher proportion of cerebrovascular disease and cancer (Table 2).

### Patient survival on dialysis

In the study period, death occurred in 1,683 (68%) individuals with ADPKD and 45,438 (74%) individuals with other causes of kidney failure. ADPKD was associated with reduced mortality risk in unadjusted analysis (hazard ratio [HR] 0.71, 95% CI 0.68–0.75). In adjusted analysis (age, gender, ethnicity smoking status, BMI, medical comorbidities, dialysis modality and dialysis vintage), ADPKD was independently associated with a lower risk of mortality (adjusted hazard ratio [aHR] 0.71, 95% CI 0.67–0.75, *p* < 0.001, Table 3). Five-year mortality was lower for patients with ADPKD than for those with other causes of kidney failure (67.0% vs. 77.5%, respectively, *p* < 0.001). Factors associated with higher mortality included chronic lung disease, coronary artery disease, peripheral vascular disease, cerebrovascular disease, diabetes and cancer. Causes of death between the ADPKD and non-ADPKD groups were similar with respect to cardiovascular disease, infection, cancer and withdrawal. Factors associated with lower mortality on dialysis included younger age, male gender, non-white ethnicity, higher body mass index, never smokers, treatment with haemodialysis and later dialysis vintage.

**Table 3:** Cox proportional hazard models for time to death in patients receiving dialysis Unadjusted + adjusted hazard ratios + 95% CI for association between ADPKD and mortality in dialysis cohort

Effect		Unadjusted			Adjusted^#^		
		HR	95% CI	*P* value	HR	95% CI	*P* value
**Disease status**							
Non_ADPKD		Ref			Ref		
ADPKD		0.71	0.68–0.75	< 0.001	0.71	0.67–0.75	< 0.001
**Age**		1.016	1.015–1.016	< 0.001	1.016	1.016–1.017	< 0.001
**Gender**							
Male		Ref			Ref		
Female		0.95	0.93–0.97	< 0.001	1.023	1.001–1.045	< 0.05
**Ethnicity**							
White		Ref			Ref		
Non-white		0.56	0.55–0.57	< 0.001	0.738	0.72–0.75	< 0.001
**Smoking status**							
Never		Ref			Ref		
Former		1.15	1.13–1.18	< 0.001	1.04	1.01–1.06	< 0.001
Current		1.05	1.03–1.09	< 0.001	1.16	1.12–1.20	< 0.001
**BMI (**kg/m^2^)							
< 18.5		Ref			Ref		
18.5–24.9		0.86	0.81–0.90	< 0.001	0.77	0.73–0.81	< 0.001
25-29.9		0.78	0.74–0.83	< 0.001	0.70	0.66–0.74	< 0.001
> 30		0.65	0.61–0.68	< 0.001	0.65	0.62–0.69	< 0.001
**Comorbidities**							
Chronic lung disease		1.36	1.32–1.39	< 0.001	1.20	1.17–1.24	< 0.001
Coronary artery disease		1.45	1.43–1.48	< 0.001	1.16	1.13–1.18	< 0.001
Peripheral vascular disease		1.53	1.49–1.56	< 0.001	1.21	1.18–1.24	< 0.001
Cerebrovascular disease		1.42	1.38–1.46	< 0.001	1.15	1.12–1.19	< 0.001
Cancer		1.22	1.19–1.25	< 0.001	1.14	1.11–1.17	< 0.001
Diabetes		1.10	1.08–1.12	< 0.001	1.29	1.26–1.32	< 0.001
**Dialysis modality**							
Haemodialysis		Ref			Ref		
Peritoneal dialysis		1.10	1.08–1.12	< 0.001	1.05	1.03–1.07	< 0.001
**Dialysis vintage**							
1989–1998		Ref			Ref		
1999–2008		0.81	0.80–0.83	< 0.001	0.84	0.82–0.87	< 0.001
2009–2018		0.58	0.57–0.60	< 0.001	0.65	0.63–0.67	< 0.001
2019–2021		0.17	0.16–0.19	< 0.001	0.24	0.22–0.27	< 0.001

Abbreviations: BMI = body mass index, KRT = kidney replacement therapy; NA = not applicable

^**#**^ age, gender, ethnicity smoking status, BMI, medical comorbidities, dialysis modality and dialysis vintage

### Transplantation

Across the study period, death occurred in 1,441(40%) transplant recipients with ADPKD and 11,036 (42%) individuals with other causes of kidney failure. In unadjusted analysis, the mortality risk was greater in the ADPKD transplant cohort (HR 1.11, 95% CI 1.05–1.17). However, in adjusted analysis (recipient age, gender, ethnicity, smoking status, BMI, medical comorbidities dialysis modality, dialysis vintage, donor source, cold ischaemic time and transplant era), there was no significant difference in mortality (aHR 0.96, 95% CI 0.88–1.04). Mortality censored for graft loss was lower in the ADPKD cohort (HR 0.81, 95% CI 0.72–0.91). Five-year mortality was similar for patients with ADPKD and other causes of kidney failure (21.4% vs. 21.5%, respectively, p value = 0.91). Factors significantly associated with mortality included age, smoking status, chronic lung disease, coronary artery disease, peripheral vascular disease, cerebrovascular disease, diabetes, cancer, prior peritoneal dialysis and donor age (Table 4). Mortality was similar in the ADPKD vs. non-ADPKD cohorts for cardiovascular disease, infection, cancer, and dialysis withdrawal.

**Table 4:** Cox proportional hazard model for time to death in transplant cohort

Effect	Unadjusted		Adjusted^#^	
	HR	95% CI	HR	95% CI
**Disease status**				
Non_ADPKD	Ref		Ref	
ADPKD	1.11***	1.05–1.17	0.96	0.88–1.04
**Recipient age**	1.036***	1.035–1.038	1.052***	1.050–1.055
**Recipient gender**				
Male	Ref		Ref	
Female	0.96	0.93-1.00	0.94*	0.88-1.00
**Recipient Ethnicity**				
White	Ref		Ref	
Non-white	0.45***	0.43–0.48	0.69***	0.65–0.74
**Recipient Smoking status**				
Never	Ref		Ref	
Former	1.64***	1.56–1.73	1.15***	1.08–1.22
Current	1.75***	1.63–1.88	1.69***	1.56–1.83
**Recipient BMI (**kg/m^2^)				
< 18.5	Ref		Ref	
18.5–24.9	1.48***	1.32–1.65	0.60***	0.53–0.68
25-29.9	1.93***	1.72–2.16	0.62***	0.55–0.71
> 30	2.20***	1.94–2.46	0.73***	0.64–0.84
**Recipient comorbidities**				
Chronic lung disease	1.89***	1.68–2.12	1.19	0.98–1.27
Coronary artery disease	2.81***	2.61–3.07	1.26***	1.16–1.38
Peripheral vascular disease	2.98***	2.7–3.29	1.38***	1.23–1.56
Cerebrovascular disease	2.58***	2.29–2.92	1.39***	1.21–1.59
Cancer	1.46***	1.35–1.57	1.22****	1.10–1.36
Diabetes	2.67***	2.53–2.82	2.04***	1.90–2.19
**Dialysis modality**				
Haemodialysis	Ref		Ref	
Peritoneal dialysis	0.99	0.95–1.03	1.068*	1.00-1.13
Pre-emptive transplant	0.44***	0.40–0.48	0.85*	0.75–0.97
**Dialysis vintage (years)**				
1989–1998	Ref		Ref	
1999–2008	0.45***	0.43–0.47	0.73***	0.67–0.80
2009–2018	0.28***	0.25–0.30	0.63***	0.54–0.74
2019–2021	0.03***	0.009–0.145	0.21	0.03–1.56
**Donor source**				
Deceased	Ref		Ref	
Live donor	0.44***	0.42–0.46	0.99	0.90–1.10
**Donor age**	0.994***	0.993–0.995	1.007***	1.005–1.009
**Cold ischaemia time (hours)**	1.037***	1.035–1.040	1.016***	1.01–1.022
**Transplant era**				
1989–1998	Ref		Ref	
1999–2008	0.49***	0.46–0.51	0.87**	0.80–0.95
2009–2018	0.34***	0.32–0.36	0.62***	0.54–0.71
2019–2021	0.13***	0.10–1.69	0.35***	0.26–0.47

Abbreviations: BMI = body mass index, KRT = kidney replacement therapy; NA = not applicable; Significance level: *<0.05, **<0.01, ***<0.001

**#** recipient age, gender, ethnicity, smoking status, BMI, medical comorbidities dialysis modality, dialysis vintage, donor source, cold ischaemic time and transplant era

### Kidney graft survival

**Fig. 1:**
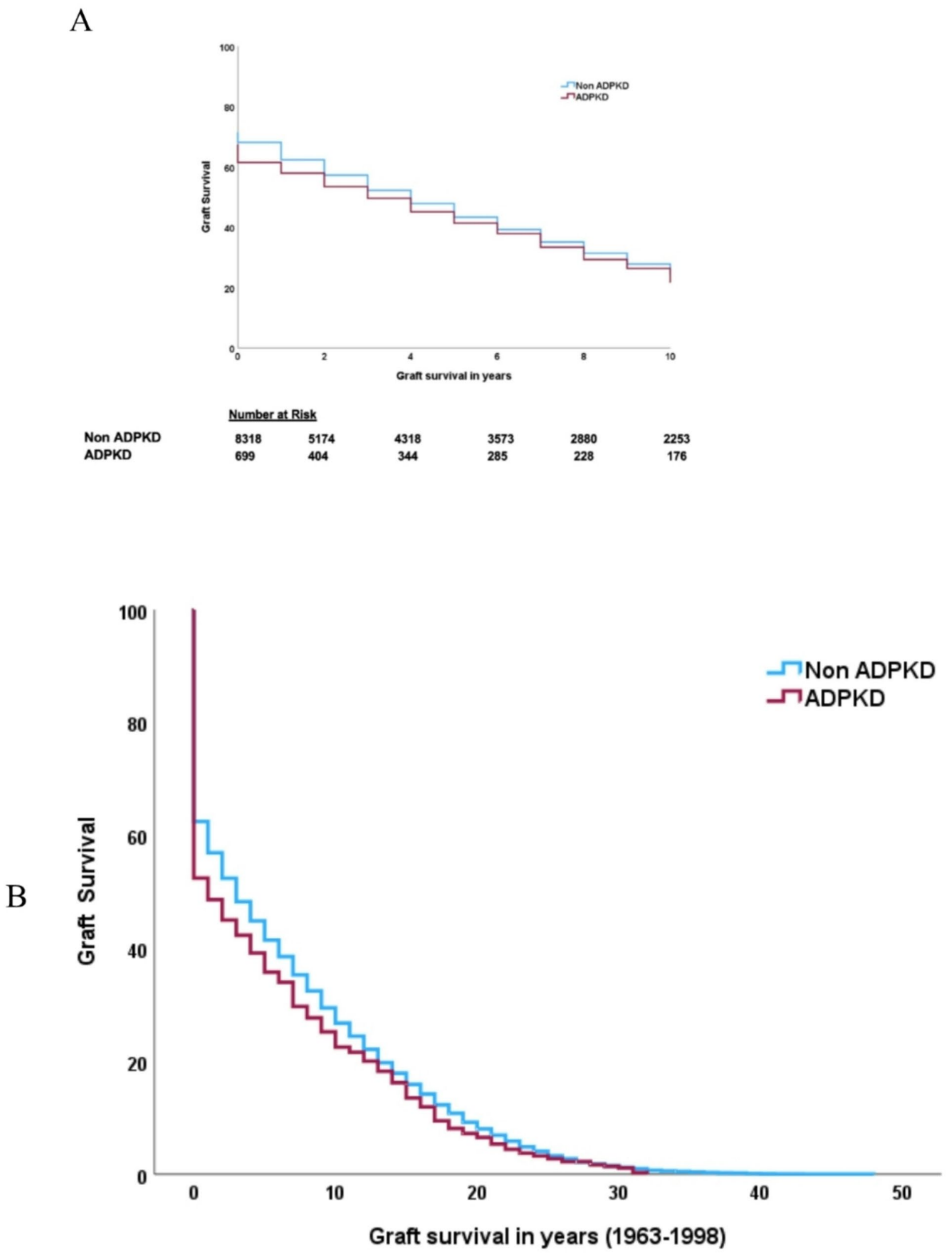

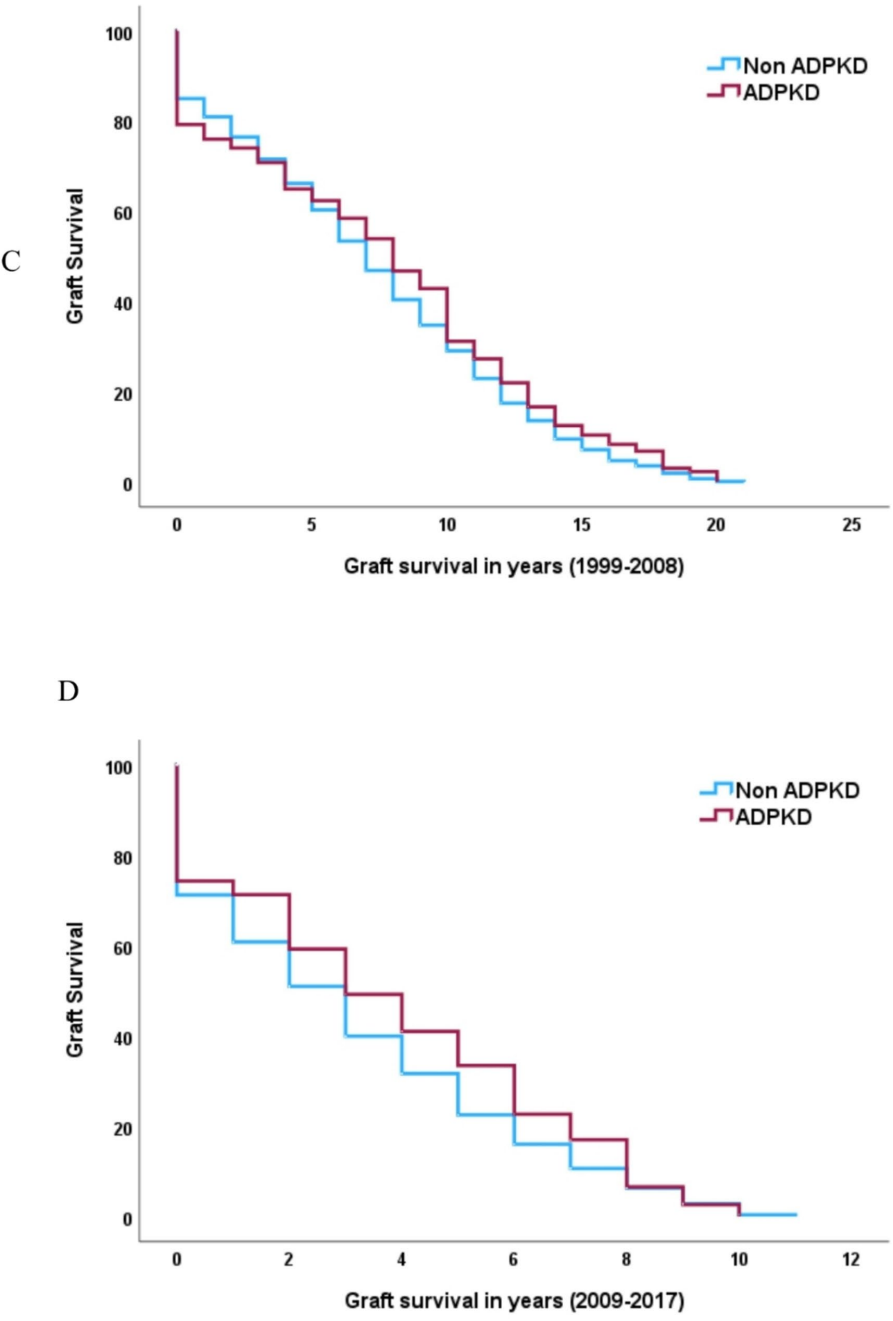
Unadjusted Kaplan Meier curve for patient survival graft failure in transplant cohort (**A**) total cohort and in (**B**) early (1963–1998) (**C**) middle (1999–2008) and (**D**) late transplant era (2009–2017)

**Fig. 2:**
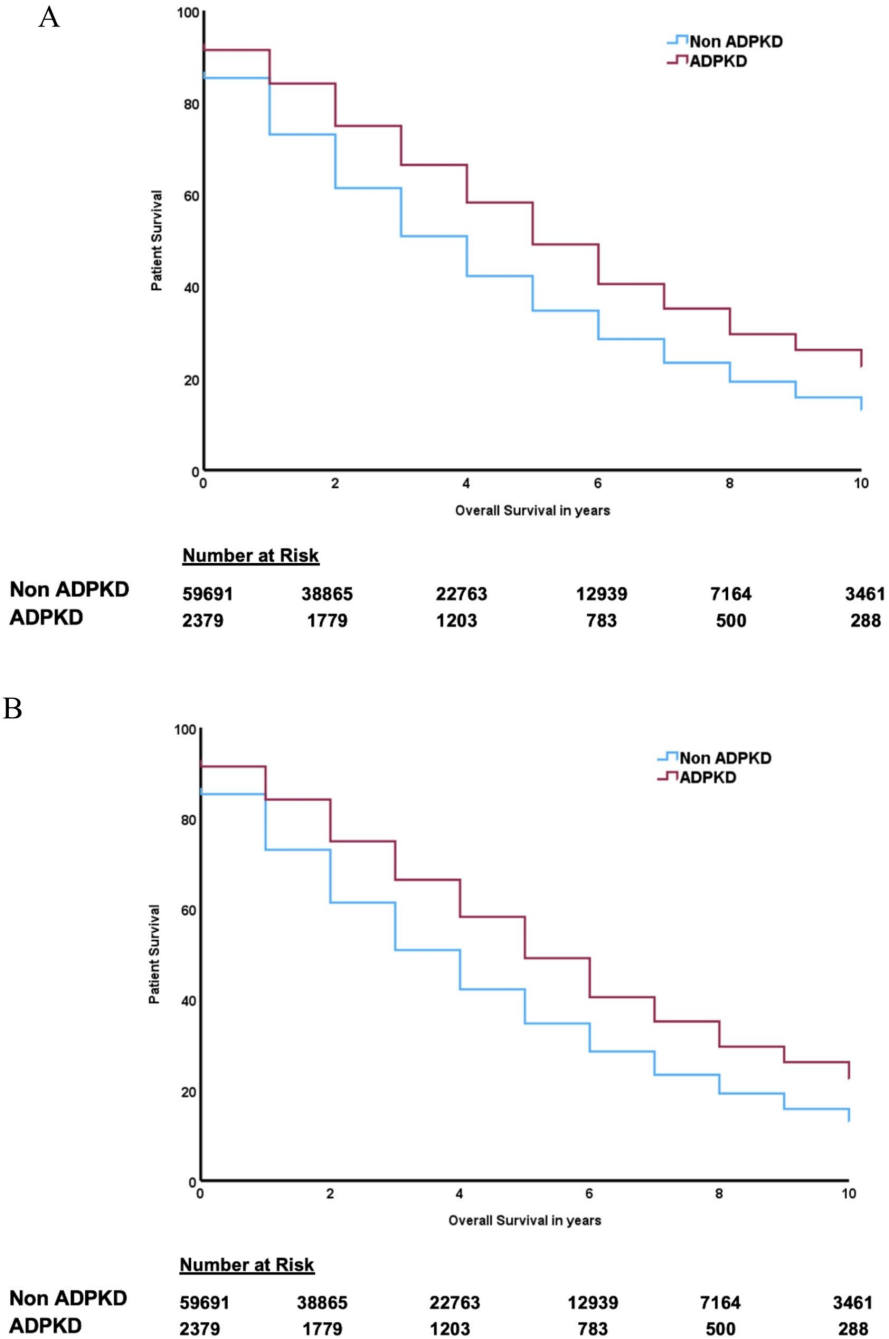
Unadjusted Kaplan Meier curves for all-cause patient survival after starting kidney replacement therapy– (**A**) dialysis and (**B**) kidney transplant

In the study period, graft failure occurred in 684 (19%) transplant recipients with ADPKD and in 11,772 (45%) transplant recipients with other causes of kidney failure. Graft failure was not different between ADPKD and non-ADPKD cohorts in unadjusted analysis, (HR 1.06, 95% CI 0.98–1.1). In adjusted analysis (recipient age, gender, ethnicity, smoking status, BMI, comorbidities, dialysis modality, dialysis vintage, donor source, donor age, cold ischaemia time and transplant era), ADPKD was associated with lower risk of graft failure (aHR, 0.87, 95% CI 0.77–0.97, *p* < 0.001, Table 5). The time to graft failure was similar between ADPKD and non-ADPKD cohorts (3 years IQR [3–10] vs. 4 years (IQR 4–10), *p* = 0.015). Graft failure whereby death was considered a competing event was similar between the ADPKD and non-ADPKD cohort, with an adjusted censored HR of 0.92, 95% CI 0.79–1.07), Table 6. Graft failure rates were lower in the ADPKD cohort compared to non-ADPKD cohort at one, three- and five-years post-transplant. Early graft failure decreased in both groups in later eras (Fig. 1). Causes of graft failure were markedly different between the ADPKD and non-ADPKD cohort, with the ADPKD cohort experiencing a higher degree of acute and hyperacute rejection (24.6% vs. 19.6%), similar rates of vascular (8.5% vs. 7.05) and technical (2.9% vs. 2.7%) graft failure with a lower incidence of chronic allograft nephropathy (8.5% vs. 51.5%), glomerulonephritis (1% vs. 5.8%) and non-compliance (1.0% vs. 3.3%) in the ADPKD transplant cohort. Factors associated with graft failure in the ADPKD cohort included increasing age, current smoker, lower BMI, peripheral vascular disease and cerebrovascular disease, later dialysis vintage, and later transplant era (Fig. 2).

**Table 5:** Cox proportional hazard model for time to graft failure in transplant cohort

Effect	Unadjusted			Adjusted^#^		
	HR		95% CI	AHR		95% CI
**Disease status**						
Non-ADPKD	Ref			Ref		
ADPKD	1.06		0.98–1.15	0.87*		0.77–0.97
**Recipient age**	1.012***		1.011–1.014	1.005***		1.002–1.007
**Recipient Gender**						
Male	Ref			Ref		
Female	0.98		0.94–1.03	0.99		0.93–1.06
**Recipient Ethnicity**						
White	Ref			Ref		
Non-white	0.92***		0.88–0.96	1.009		0.94–1.09
**Smoking status**						
Never	Ref			Ref		
Former	1.12***		1.06–1.19	1.02		0.95–1.09
Current	1.21***		1.12–1.31	1.13**		1.04–1.24
**BMI (**kg/m^2^)						
< 18.5	Ref			Ref		
18.5–24.9	1.07		0.98–1.17	0.90*		0.81-1.00
25-29.9	1.19***		1.08–1.31	0.88*		0.79–0.99
> 30	1.46***		1.31–1.62	0.98		0.87–1.12
**Recipient comorbidities**						
Chronic lung disease	1.41***		1.21–1.65	1.06		0.89–1.27
Coronary artery disease	1.57***		1.41–1.75	1.12		0.98–1.27
Peripheral vascular disease	1.75***		1.52–2.02	1.20*		1.00-1.43
Cerebrovascular disease	1.59***		1.34–1.89	1.29**		1.06–1.58
Cancer	1.09		0.97–1.23	1.08		0.92–1.29
Diabetes	1.42***		1.33–1.53	1.04		0.94–1.14
**Dialysis modality**						
Haemodialysis	Ref			Ref		
Peritoneal dialysis	1.01		0.97–1.06	1.03		0.96–1.10
Pre-emptive transplant	0.80***		0.73–0.88	0.92		0.82–1.03
**Dialysis vintage (years)**						
1989–1998	Ref			Ref		
1999–2008	1.06*		1.01–1.13	1.11*		1.00-1.24
2009–2021	1.83***		1.68–1.99	1.54***		1.20–1.71
**Donor source**						
Deceased	Ref			Ref		
Live donor	0.77***		0.73–0.81	0.91		0.81–1.01
**Donor age**	1.002*		1.000-1.003	1.006***		1.004–1.008
**Cold ischaemia time (hours)**	1.005***		1.002–1.008	1.004		0.99–1.01
**Transplant era**						
1989–1998	Ref			Ref		
1999–2008	0.99		0.94–1.04	1.41***		1.28–1.56
2009–2021	1.70***		1.59–1.82	2.31***		1.98–2.75

Abbreviations: AHR = adjusted hazard ratio, BMI = body mass index, KRT = kidney replacement therapy; NA = not applicable; Significance level: *<0.05, **<0.01, ***<0.001

**#** recipient age, gender, ethnicity, smoking status, BMI, comorbidities, dialysis modality, dialysis vintage, donor source, donor age, cold ischaemia time and transplant era

**Table 6:** Graft failure censored mortality risk and death as a competing for graft failure in the transplant cohort

Effect	Mortality			Graft Failure		
	ASHR		95% CI	ASBMcoHR		95% CI
**Disease status**						
Non-ADPKD	Ref			Ref		
ADPKD	0.81***		0.72–0.91	0.92		0.79–1.07
**Recipient age**	0.997*		0.994–0.999	1.035***		1.031–1.039
**Recipient Gender**						
Male	Ref			Ref		
Female	1.02		0.96–1.08	1.037		0.94–1.13
**Recipient Ethnicity**						
White	Ref			Ref		
Non-white	1.10**		1.03–1.17	0.73***		0.66–0.80
**Smoking status**						
Never	Ref			Ref		
Former	1.14***		1.06–1.22	1.12*		1.02–1.24
Current	1.46***		1.33–1.59	1.45***		1.28–1.64
**BMI (**kg/m^2^)						
< 18.5	Ref			Ref		
18.5–24.9	0.90		0.81-1.00	0.66***		0.56–0.78
25-29.9	1.08		0.96–1.22	0.65***		0.54–0.78
> 30	1.21**		1.07–1.38	0.78*		0.64–0.94
**Recipient comorbidities**						
Chronic lung disease	1.08		0.90–1.30	1.17		0.91–1.49
Coronary artery disease	1.23**		1.08–1.40	1.23**		1.05–1.43
Peripheral vascular disease	1.16		0.97–1.38	1.28**		1.04–1.57
Cerebrovascular disease	1.43***		1.17–1.74	1.37**		1.08–1.74
Cancer	0.94		0.80–1.11	1.01		0.88–1.36
Diabetes	1.60***		1.46–1.75	1.55***		1.38–1.74
**Dialysis modality**						
Haemodialysis	Ref			Ref		
Peritoneal dialysis	1.05		0.98–1.12	1.07		0.97–1.19
Pre-emptive transplant	1.10		0.98–1.23	0.81*		0.66–0.99
**Dialysis vintage (years)**						
1989–1998	Ref			Ref		
1999–2008	1.78***		1.59–1.99	0.71***		0.61–0.83
2009–2021	4.2***		3.51–5.12	0.53***		0.40–0.71
**Donor source**						
Deceased	Ref			Ref		
Live donor	1.06		0.95–1.18	0.85		0.72-1.00
**Donor age**	1.017***		1.015–1.019	1.004**		1.002–1.007
**Cold ischaemia time (hours)**				1.014**		1.005–1.023
**Transplant era**						
1989–1998	Ref			Ref		
1999–2008	1.14**		1.02–1.26	1.18**		1.04–1.35
2009–2021	0.84*		0.71–0.99	1.50***		1.18–1.92

Abbreviations: AHR = adjusted hazard ratio, BMI = body mass index, KRT = kidney replacement therapy; NA = not applicable; Significance level: *<0.05, **<0.01, ***<0.001

## Discussion

This retrospective, bi-national, registry-based study investigated the characteristics and clinical outcomes of people kidney failure due to ADPKD commencing KRT in Australia and New Zealand. Compared to people with other causes of kidney failure, the ADPKD cohort had better survival outcomes and lower medical comorbidities at initiation of dialysis. For those receiving a kidney transplant, patient survival was similar between the ADPKD and non-ADPKD cohorts. Graft survival was better in the ADPKD cohort in adjusted analysis, graft-survival rates were similar in death censored analysis. Cardiovascular disease was present in one quarter of patients with ADPKD at initiation of KRT and was the most common cause of death in all KRT.

The key finding from this study is that survival on dialysis in the ADPKD cohort in adjusted analysis was superior to that of the non-ADPKD cohort. Causes of death were similar between ADPKD and other causes of kidney failure and cardiovascular disease was the main cause of mortality in both cohorts. Our findings are consistent with international data which demonstrate survival advantage in ADPKD, including a large USA based dialysis KRT study which also found better survival in 10,178 ADPKD patients on dialysis compared to nondiabetic controls [15]. European registry data including 35,164 patients with ADPKD undergoing KRT found improved 2-year survival on dialysis compared to kidney failure from other causes, with a reduction in cardiovascular deaths over time in the ADPKD cohort [16]. ADPKD patients also have improved survival in Spanish KRT cohorts and French peritoneal dialysis ADPKD cohorts [17, 18]. Of note, initial KRT modality with peritoneal dialysis in our cohort was associated with increased mortality compared to haemodialysis, consistent with evidence suggesting peritoneal dialysis may be associated with increased risk of cardiovascular mortality after one year, though this is not consistent in all studies [19, 20]. This study is a retrospective, observational study, where each groups has inherent differences which may not be accounted for, including switching dialysis modality or transplantation; a study which propensity matched haemodialysis and peritoneal dialysis groups found similar survival outcomes [21]. Previous ANZDATA registry studies found reduced mortality in patients with polycystic kidney disease with improved survival in modern dialysis eras, in keeping with global data [9, 22]. Overall our data are consistent with international studies in KRT that ADPKD confers a survival advantage in those with kidney failure requiring dialysis.

Improved survival outcomes in patients with ADPKD on dialysis compared to those with kidney failure from other causes may reflect several factors unique to ADPKD populations. Firstly, the lower burden of medical comorbidities, reduced levels of smoking and younger age at onset of KRT are all factors associated with improved survival, and are more common in the ADPKD cohort. Commencing dialysis at a younger age is associated with improved survival in KRT which may include increased rates of kidney transplantation [23]. Secondly, there may be unique health factors associated with ADPKD that contribute to differences in mortality. ADPKD is a multi-system disease associated with intracranial aneurysm, diverticulitis, polycystic liver disease and valvular heart disease which have potential to impact mortality. However, a large case control series of ADPKD patients found that deaths related to cardiac valvular disease and cerebrovascular disease were lower in ADPKD patients, with similar mortality bowel perforation and vascular haemorrhage, with only polycystic liver disease morbidity increased in the ADPKD cohort [15]. The survival advantage in ADPKD patients on dialysis persists even with these medical comorbidities. Potentially, a clinical diagnosis of ADPKD may enable screening and early recognition of potential complications such as intracranial haemorrhage, although further research to understand these trends are required [24]. Thirdly, global changes in disease management and trends may significantly impact ADPKD patients resulting in improved mortality outcomes. Notably, the cause of death in ADPKD cohorts has changed significantly over time, with data showing less deaths from infection and increasing deaths from cardiovascular disease, with more recent trends highlighting increasing cardiac deaths from ischaemic heart disease and cardiac hypertrophy in ADPKD populations [10, 25]. Improved infectious outcomes in ADPKD may be related to changes in management overtime including imaging and use of lipid-soluble antibiotics [26]. Changes in cardiovascular disease in ADPKD are uncertain but may related to use of anti-hypertensive agents including renin angiotensin blockade and paradoxical increase in coronary artery disease due to longer survival on KRT, but data to support this are lacking [27]. Increased use of anti-hypertensives may also have contributed to improved outcomes in ADPKD cohorts, although uptake in some cohorts was as low as 37.3% [28–30]. Fourthly, unmeasured socioeconomic and environmental factors which may also contribute to reduced mortality in ADPKD cohorts. Some studies suggest patients with ADPKD are motivated to adopt lifestyle measures such as salt reduction, exercise, hypertension management, which may be distinct from other causes of kidney failure [31]. An important but unmeasurable factor unique to ADPKD is the potential significance that family history and experience may have on patients personal understanding and experience with kidney failure and motivation for treatment. To summarise, there are likely many complex social and medical factors that contribute to improved mortality in ADPKD patients that warrant further investigation to potentially guide strategies to improve outcomes.

This study found transplant patient survival was similar amongst patients with ADPKD and other causes of kidney failure. Cardiovascular disease and infection were the most significant causes of mortality in both transplant cohorts. The burden of comorbidities at time of transplant was distinctly different between the ADPKD cohort compared to other causes of kidney failure, with less chronic lung disease, peripheral vascular disease, and diabetes, but increased cerebrovascular disease and cancer, and similar incidence of cardiovascular disease, all of which were associated with increased mortality risk, particularly diabetes. Single centre and registry data with extended follow up also found similar survival outcomes between patients with ADPKD and those with other causes of kidney failure, with some studies suggesting this may be related to lower incidence of coronary artery disease and more pre-emptive kidney transplants [15, 32–36]. European registry studies with shorter follow up periods (2–3 years), found that survival in ADPKD transplant recipient was improved compared with non-ADPKD cohorts, suggesting there may be an early survival advantage, although the one- and three-year mortality in this study were similar between groups in our study [16, 17]. Our data are in keeping with global long term registry data, that ADPKD patients have similar transplant survival to those other causes of kidney failure, despite the survival advantage seen in the ADPKD dialysis cohorts. Given transplant patients are subject to stringent selection criteria, and the generally low degree of medical comorbidities across transplant cohorts, this may explain similar survival outcomes in the transplant population. The survival advantage seen in ADPKD transplant recipients in smaller studies may reflect differences in local practice including lower rates of cardiovascular disease. That ADPKD patients have similar transplant survival to other causes of kidney failure likely reflects the selection process all transplant undergo, and it is reassuring to see similar survival to their peers.

Graft survival was better in the ADPKD cohort in the adjusted risk analysis for kidney transplant survival, however there was no advantage in death-censored graft survival. This suggests that there is no survival advantage in ADPKD cohorts when overall patient survival is considered. Notably, graft survival in both cohorts was reduced in later eras when compared to earlier eras, which may reflect changing trends in transplant recipients including greater numbers of patients transplanted, but warrants further exploration. Acute and hyperacute rejection were the most common causes of transplant failure in the ADPKD cohort, with markedly lower incidence of recurrent nephropathy, as expected in a genetic rather than immune mediated kidney disease. Globally, transplant outcomes in ADPKD cohorts are variable, with some cohorts reported to have increased risks of rejection, diabetes, thromboembolism and metabolic complications, whilst other cohorts were found to have improved graft survival with similar rates of rejection [32, 37, 38]. Given the increased propensity of cyst complications in ADPKD clinicians may elect to reduce immunosuppression in the setting of urological infections which could contribute to transplant rejection. A retrospective case control series identified that ADPKD transplant recipients with higher blood levels of mycophenolate were associated with increased risk of urinary tract infections [39]. Further research is needed to understand and improve outcomes in ADPKD recipients undergoing transplant with a focus on understanding factors associated with infection and rejection.

Cardiovascular and cerebrovascular diseases are significant contributors to morbidity and mortality in ADPKD patients receiving KRT which may be related to intrinsic disease processes in ADPKD, hypertension and risk driven by kidney failure. Cardiovascular disease is prevalent in the ADPKD cohort, and while this is lower than their peers with kidney failure from other causes, is still the most common cause of mortality, in keeping with international data [15, 16]. ADPKD is associated with increased risk of intracranial aneurysms, with current guidelines recommending screening in at risk patients [40]. Cerebrovascular disease can be aneurysmal rupture, ischaemic stroke or hypertensive stroke, with hypertension representing a predominant risk factor in ADPKD [3, 41]. In our ADPKD cohort 3.7% had cerebrovascular disease at commencement of KRT, although ANZDATA is unable to distinguish ischaemic from haemorrhagic stroke. Data linkage studies have found an increased incidence of both ischaemic and haemorrhagic stroke in ANZDATA KRT cohort. Further work is required to understand burden and risk of ischaemic stroke in the ADPKD cohort, which may be under recognised.

The strengths of this study include a large study population with all incident patients commencing KRT in Australia and New Zealand with longitudinal follow up and assessment. Statistical analyses were able to account of many important patient and centre-level characteristics. This study has limitations including presence of unmeasured confounders and limited details on types of cardiovascular disease and distinction of forms of cerebrovascular disease. This is a voluntary data set with no external validation process, and is at risk of coding bias.

## Conclusions

This study found that KRT patients in Australian and New Zealand have improved mortality and fewer comorbidities in the dialysis cohort and with similar transplant survival and death-censored graft survival compared to their peers.

## Data Availability

No datasets were generated or analysed during the current study.

## Abbreviations

ADPKD: Autosomal dominant polycystic kidney disease
KRT: Kidney replacement therapy
aHR: Adjusted hazard ratio
CI: Confidence interval
